# A new Maxwell model and its application in researching the compressive creep properties of recombinant bamboo

**DOI:** 10.1371/journal.pone.0329095

**Published:** 2025-07-28

**Authors:** Shanshan Shen, Qifeng Gao, Xiaolin Gong, Songsong Sun, Jiahong Fu

**Affiliations:** 1 College of Mechanical and Electrical Engineering, Zhejiang Industry Polytechnic College, Shaoxing, Zhejiang, China; 2 College of Automobile and Traffic Engineering, Nanjing Forestry University, Nanjing, Jiangsu, China; 3 College of Engineering, Hangzhou City University, Hangzhou, China; Southwest University of Science and Technology, CHINA

## Abstract

Fiber-reinforced composite materials manufactured through gluing techniques, such as recombinant bamboo, usually exhibit obvious creep behavior during service. In this work, the compressive creep performance of the recombinant bamboo was selected as the research object. First, a compressive fracture experiment was proposed to determine the compressive strength of the material. Second, different groups of compressive creep experiments were carried out on four samples under several stress levels. Finally, the new Maxwell model proposed in this paper was selected for analyzing the creep performance of the material. The main conclusion drawn from the research is that, compared with traditional commonly used models (Kelvin and Burgers), the newly proposed Maxwell model, which is based on the theory of the variable-order fractional derivative, can more accurately simulate the compressive strain creep growth property with relatively fewer parameters, and the stress level effect on the main model parameters can be accurately determined, which makes this approach valuable for actual engineering applications.

## 1. Introduction

Bamboo is a typical natural material widely planted around the world. Compared with wood materials, bamboo materials have several advantages, such as lower cost, faster growth speed and good mechanical properties, which make them widely applied in modern industry [1]. On the other hand, the most commonly application conditions of the bamboo materials are usually the construction and furniture industry. As a result of this, correct evaluation of the material mechanical property becomes indispensable before the application [2,3].

In recent years, a large amount of work has been carried out on researching the mechanical property of the bamboo materials. Among these Huang researched the bending fracture behavior of recombinant bamboo via different beam models; in this way, several important parameters of the mechanical properties of the material can be calculated [4,5]. Li researched the axial mechanical properties of recombinant bamboo in different fiber directions and proposed corresponding empirical equations for accurately fitting the stress‒strain relationship. In addition, the fiber direction clearly affects the mechanical properties [6]. Ma researched the bending fatigue property of recombinant bamboo and reported that the high cycle bending fatigue life of this material could reach as high as 10^6^. In addition, the residual stiffness of the material was obviously influenced by the stress amplitude applied to the sample [7]. Shangguan conducted corresponding research on the influence of heat treatment on mechanical properties, and the results revealed that the material strength was affected by the temperature during the treatment process, which was obviously due to the phenolic resin solidification phenomenon, as well as the crystallinity of cellulose [8]. Yu reported that increasing pressure during the manufacturing process can obviously improve the strength of a material, which can be explained by enhancing the interfacial properties between the fiber and the matrix, as well as the density of the sample [9]. Yuan chose the influence of the hot oil bath technique on the material strength to be the research object and reported that the strength reduction caused by this approach would occur only if the bath time reached a certain value, which could be attributed to enough oil molecules invading the fiber‒matrix interface [10].

According to earlier studies, recombinant bamboo was usually considered a typical type of anisotropic elastic composite material [11]. According to the theory of viscoelastic mechanics, both the fiber and the matrix of a material usually exhibit obvious creep behavior when an external load is applied. Accordingly, corresponding work has been conducted in recent years. Wei conducted different kinds of standard creep experiments (tensile, compressive, and bending) on recombinant bamboo and reported that the creep process of the material was clearly affected by both the load type and the stress level. In addition, the classical Burgers model can accurately simulate the short-term creep strain growth property [12–15]. Liu researched the creep performance of recombinant bamboo via different models and reported that, compared with the Burgers model, the proposed five-parameter model with a modification factor could accurately simulate the nonlinear viscous component of the creep strain [16]. Liu also proposed an accelerated creep experiment method for recombinant bamboo based on short-term creep experiments under variable temperature conditions; in this way, long-term creep performance prediction of the material could be achieved to shorten the experimental process [17].

In a previous study, the fractional derivative theory was applied to research the tensile creep performance of recombinant bamboo. The results showed that compared with traditional models, modified models (Maxwell and Kelvin) defined by the selected fractional derivative approach can better express the creep strain growth property of a material [18,19]. According to published papers, the compressive mechanical properties of recombinant bamboo are quite different from those of tensile bamboo. Thus, whether these models are suitable for researching the compressive creep performance of a material is still unknown [6]. In addition, although the stress level of the creep experiment clearly affects the main parameters of the model, the detailed influence information could not be expressed clearly due to the dispersion of the material properties. As a result, a quantitative analysis of the creep performance was also difficult to perform.

In this paper, a modified Maxwell model based on the variable-order fractional derivative approach is proposed for researching the compressive creep performance of recombinant bamboo. First, a standard compressive fracture experiment was conducted to determine the corresponding strength of the material. Second, a compressive creep experiment under a time-varying load was carried out to record the creep strain evolution process. Finally, different kinds of models were selected for analyzing the viscoelastic properties of the material. Compared with traditional commonly used models (Kelvin and Burgers), the proposed new Maxwell model, which is defined based on the theory of the variable-order fractional derivative, can more accurately simulate the compressive strain creep growth property with relatively fewer parameters. In addition, the influence of the stress level on the creep property can be accurately expressed by the selected functions. These advantages make this approach more suitable for researching the creep performance of this material, and it has very wide popularization and application prospects in modern industry.

## 2. Materials and methods

### 2.1. Material

**Fig 1:**
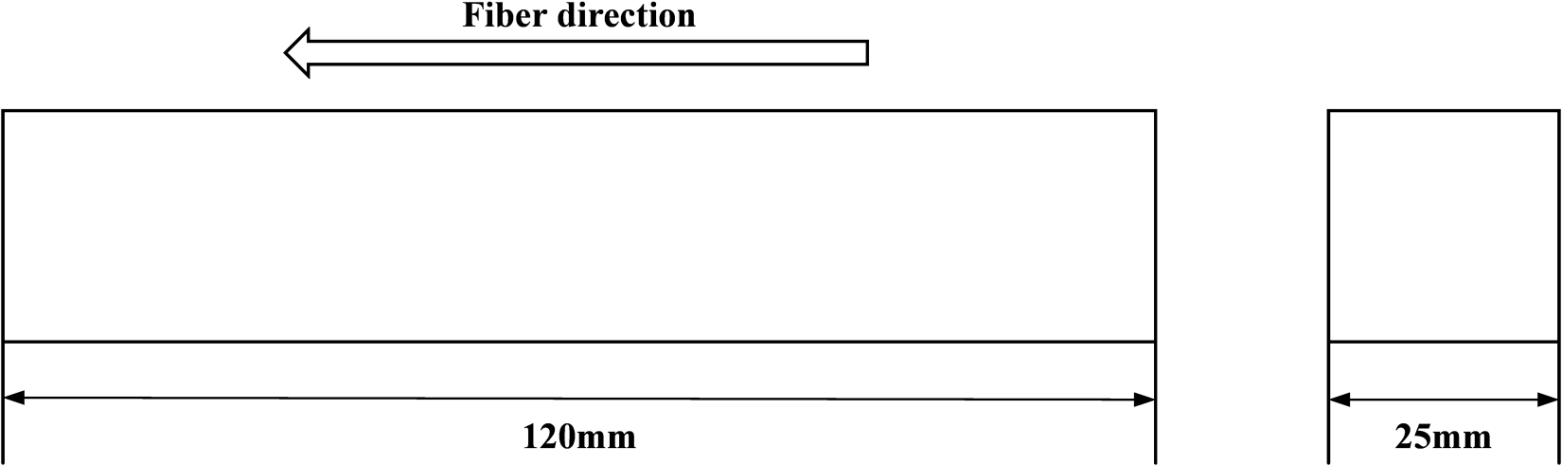
Experimental specimens.

In this work, the object of study is recombinant bamboo. Fig 1 shows the information of the main structural parameters of the sample for all the experiments proposed in this paper, from which it can be found that the shape of the sample is cuboid. The material was provided by Taohua Jiang Company, Ltd., through a hot pressing technique. During the first step of this approach, natural bamboo fibers were produced from raw bamboo material through combined physical and chemical approaches. The fibers were subsequently orderly arranged into groups and mixed with the phenolic resin. Finally, the mixture was placed into the given steel boxes and compressed under the given pressure and high enough temperature. In this way, the fibers and the phenolic resin were combined to generate a solid sample. The raw bamboo material used in this study was 5-year-old moso bamboo from Hunan Province. The density of the material is 1280 kg/m^3^. The water content of the material is 6.4%. The fiber volume fraction is greater than 70%. In addition, the ASTMD standard (the serial number is 143−94) is applied to guide the preparation of the sample [20].

### 2.2. Compressive fracture experimental methods

**Fig 2:**
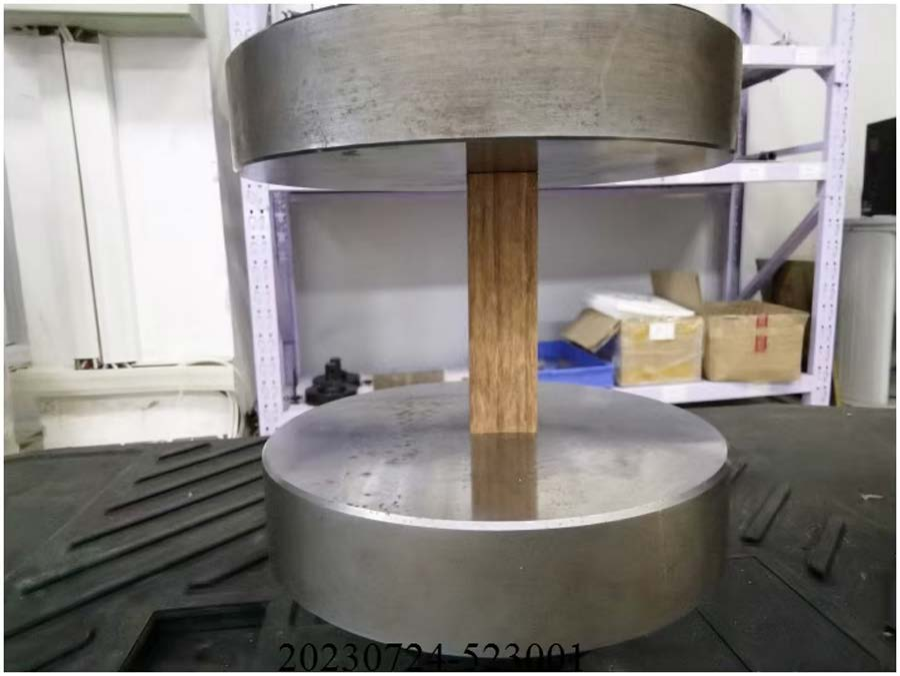
Compressive fracture experimental equipment and sample.

According to the related creep research of composite materials, during creep experiments, the stress amplitude is usually defined according to the strength of the material itself. In other words, before the creep experiment, the corresponding type of strength of the material should be determined in advance. In this work, the type of creep research is compressive. Fig 2 shows the compressive fracture experimental equipment for the recombinant bamboo, from which it can be observed that the sample was uprightly arranged on the platform. In addition, the steel head is pressed on the top face of the sample. During the compressive fracture experiment process, the amplitude of the compression load increases steadily until fracture occurs. All the experimental equipment and corresponding operation work were provided by the STANDARD Testing Group Co., Ltd. In addition, owing to the obvious dispersion of the material properties, the sample size of the experiment should be large enough. In this work, eleven groups of experiments were carried out. The standard with the serial number GB T1041-2008 was chosen to guide the compressive experiment operation process in this paper [21].

### 2.3. Compressive creep experimental methods

When the compressive strength of the material is determined, the key parameter in guiding the creep experiment can also be proposed. According to previous studies, for compressive creep experiments on bamboo materials, the stress level should usually be no more than 40% of the compressive strength to avoid quick breakage or fracture of the sample. However, for the creep experiments conducted on different samples, the creep strain values are usually also quite different from each other even though the stress level applied on them is the same. The main reason for this phenomenon is the uneven microstructure features of the samples, as well as the dispersion of the material properties. As a result, accurate analysis of the creep performance and model verification is difficult.

**Fig 3:**
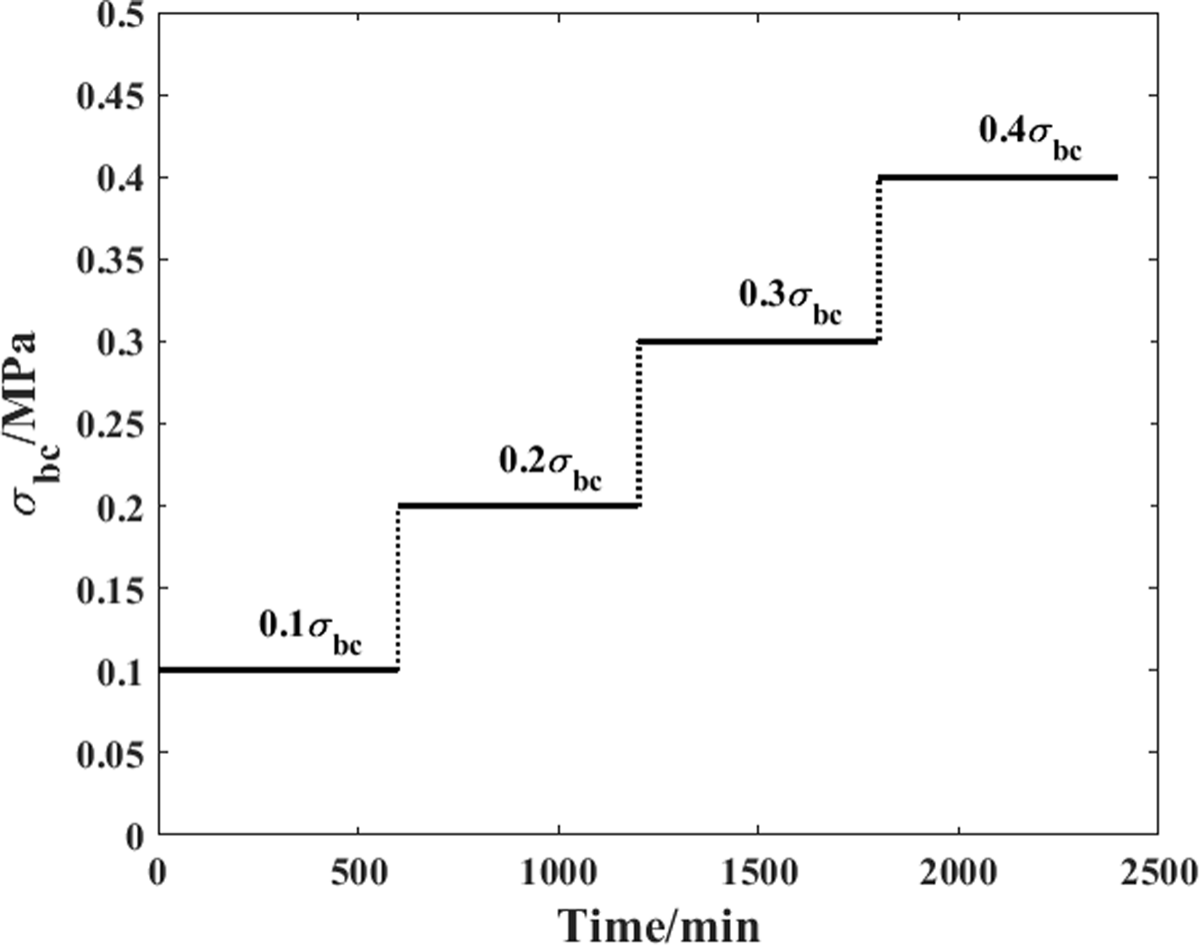
Load history of the creep test.

To overcome this problem, a time-varying compressive load is proposed in this paper. Fig 3 shows the detailed information of the load history. In the first stage of the loading process, the stress level is set to 10% of the compressive strength and lasts 10 hours to record the corresponding creep strain evolution process within this time step. In the second step, the stress level increases to 20% of the compressive strength, and the creep strain from the same sample is also recorded for further analysis. In the third and fourth steps, the values of the stress level are 30% and 40% of the compressive strength, respectively. Based on this load history, the compressive strains under different stress levels are recorded from the same sample. In this way, the dispersion effect of the material properties can be considered in advance.

**Fig 4:**
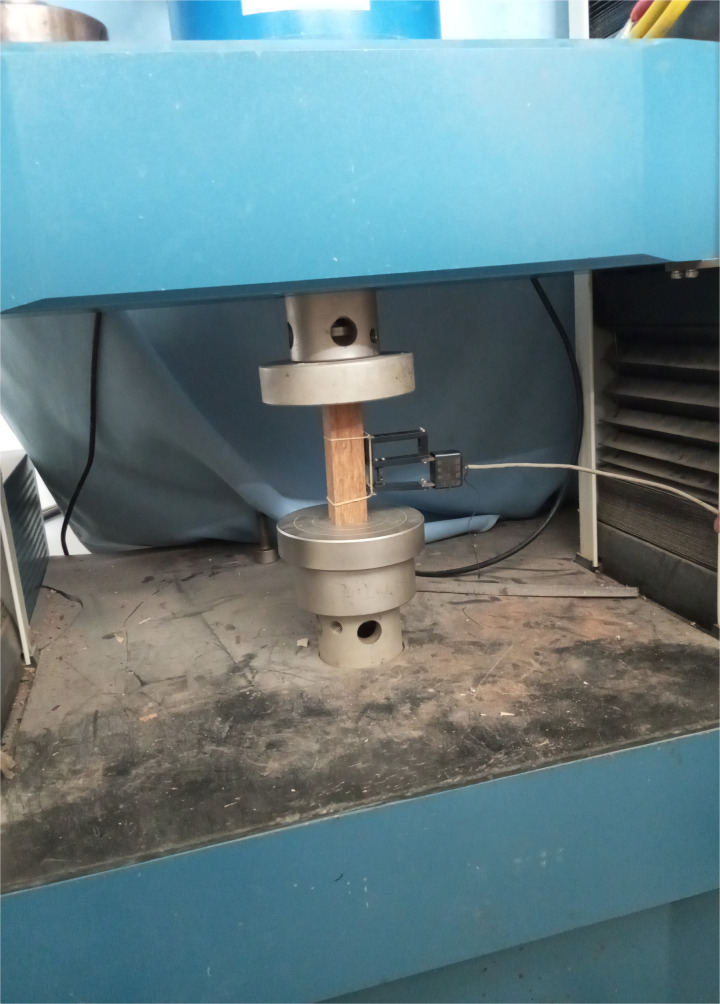
Electronic extensometer and the sample used for the compressive creep test.

As introduced above, the creep performance of a material is usually achieved by analyzing the recorded creep strain. At present, this factor is usually recorded by either the strain gap or the electronic extensometer. According to previously published papers, the electronic extensometer is superior to the strain gap under these conditions due to its greater stability. The detailed information of this equipment and the corresponding samples are shown in Fig 4.

**Fig 5:**
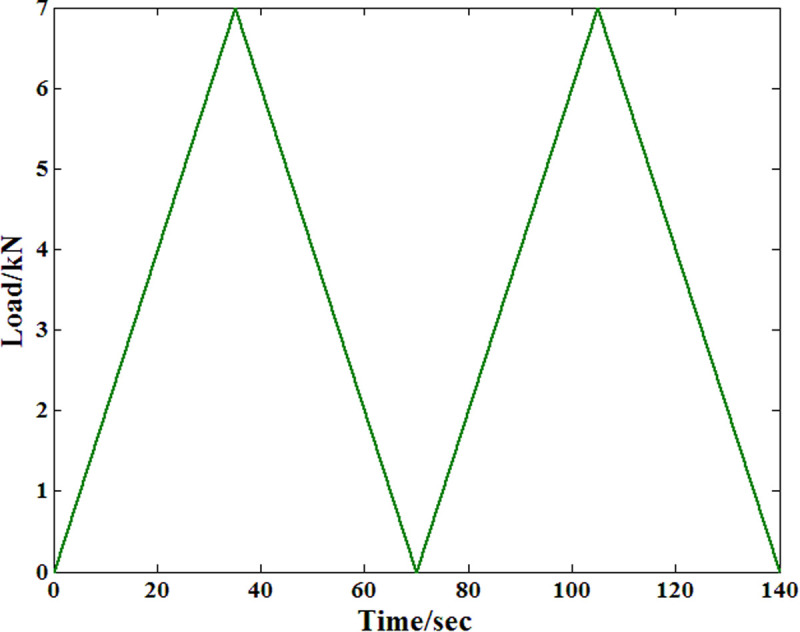
Load for the pretreatment.

As shown in Fig 4, the electronic extensometer is fixed at the middle area of the sample. In this work, the length of the electronic extensometer is 50 mm. Therefore, the value of the corresponding compressive creep strain can be determined as follows:


$$\varepsilon =\frac{\Delta L}{50}$$ (1)

where ΔL is the displacement of the electronic extensometer itself. In this way, the creep strain value can be determined by monitoring the displacement of the sample under an external load. On the other hand, other factors, such as the residual stress within the sample generated during the manufacturing process, may affect the creep strain measurement. To avoid this situation, corresponding pretreatment is necessary. The detailed information of this load is shown in Fig 5.

### 2.4. Variable-order fractional derivative method and Maxwell model

**Fig 6:**
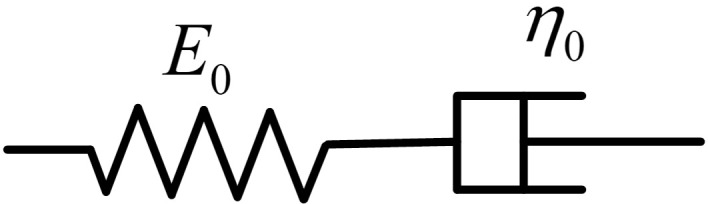
Main components of the Maxwell model.

At present, an important step in researching the viscoelastic properties of composite materials is to choose a suitable mechanical constitutive relation model to express the stress‒strain relationship of the material. In recent years, various types of models have been reported, among which the Maxwell model is considered one of the most classical and effective choices in this situation [22]. The main components of this model are shown in Fig 6.

As shown in Fig 6, the Maxwell model is composed of two parts: the spring body and the Abel dashpot body. Thus, the stress‒strain relationship of the model can be expressed as follows:


$$\sigma_{1}=\sigma_{2}=\sigma_{0}$$ (2)

$$\sigma_{1}=E_{0}\varepsilon_{1}\left(t\right)$$ (3)

$$\sigma_{2}=F(\;\;\;\begin{array}{c} {}*20c\varepsilon_{2},\eta_{0}\;\;\; \end{array})$$ (4)

$$\varepsilon_{0}=\varepsilon_{1}+\varepsilon_{2}$$ (5)

where $\sigma_{0}$ and $\varepsilon_{0}$ are the total amount of the stress and strain provided by the model, respectively; $E_{0}$ and $\eta_{0}$ are the elastic modulus of the spring body and the coefficient of viscosity of the dashpot body, respectively; F is the stress‒strain relationship function of the dashpot body; $\sigma_{1}$ and $\varepsilon_{1}$ are the stress and strain of the spring body, respectively; and $\sigma_{2}$ and $\varepsilon_{2}$ are the stress and strain of the dashpot body, respectively. At present, the stress‒strain relationship of the spring body is clear. For the dashpot body, no standard definition has been reported. In earlier studies, the dashpot body was usually considered a standard Newtonian fluid body. In actual engineering applications, the dashpot usually has an obvious viscoelastic property, which is quite different from that of a Newtonian fluid body.

On the other hand, another feature of the creep process is that the mechanical property of the material usually shows obvious time-varying characteristics. Meanwhile, the fractional order derivative approach is usually considered as a typical kind of time-varying research tool. Based on this fact, some experts have proposed that the fractional derivative approach may be a suitable choice for researching this problem. The stress‒strain relationship of the dashpot body defined by this approach can be expressed as follows:


$$\sigma_{2}=\eta_{0}\frac{d^{\alpha }\varepsilon_{2}\left(t\right)}{dt^{\alpha }}$$ (6)

As shown in Equation (6), α is the order of the fractional derivative. If the value of the order is equal to 0, the body is converted into the standard spring body. When the value of the order is 1, the body is converted into a standard Newtonian fluid body. When the value of the order is between 0 and 1, the dashpot body defined by this will show viscoelastic property, which makes it suitable for studying the creep behavior. To date, this approach has been widely applied in research on viscoelastic mechanical properties, such as creep performance. In addition, in addition to traditional creep tests, other experimental techniques, such as nanoindentation, have also been adopted in this field [23]. These approaches provide thermodynamically consistent formulations, incorporate experimental calibrations, and naturally handle uncertainty; in this way, more reliable analyses of the viscoelastic properties can be conducted. At present, there are various definition models for this theory, among which the R-L (Riemann–Liouville) fractional derivative model is usually considered an effective choice in this situation. The detailed definition of this model can be expressed as follows:


$$D^{\alpha }f\left(t\right)=\int_{0}^{t}\frac{f^{\prime }\left(\tau \right)}{\Gamma \left(1-\alpha \right){\left(t-\tau \right)}^{\alpha }}d\tau$$ (7)

where Γ is the Gamma function. According to this model, the response strain of the dashpot body under the given stress level can be determined as follows:


$$\varepsilon_{2}\left(t\right)=\frac{\sigma_{0}(t)}{\eta_{0}}\frac{t^{\alpha }}{\Gamma (\alpha +1)}$$ (8)

For the whole Maxwell model, the stress‒strain relationship can be expressed as follows:


$$\varepsilon \left(t\right)=\varepsilon_{1}\left(t\right)+\varepsilon_{2}\left(t\right)=\frac{\sigma_{0}}{E_{0}}+\frac{\sigma_{0}(t)}{\eta_{0}}\frac{t^{\alpha }}{\Gamma (\alpha +1)}$$ (9)

As shown in Fig 3, the creep load in this paper is time-varying. According to previously published papers, when the Maxwell model is adopted for researching the viscoelastic properties of a material, the values of the main model parameters are obviously influenced by the stress level. Thus, the corresponding creep strain response of the body under this load condition can be expressed as follows:


$$\varepsilon \left(t\right)=\sum_{i=1}^{n}\left[\varepsilon_{1i}\left(t\right)+\varepsilon_{2i}\left(t\right)\right]=\sum_{i=1}^{n}\left[\frac{\sigma_{0i}}{E_{0i}}+\frac{{\left(t_{i}-t_{i-1}\right)}^{\alpha_{i}}\sigma_{0i}}{\eta_{0i}\Gamma (\alpha_{i}+1)}\right]$$ (10)

As shown in Equation (10), the subscript i represents the model parameter in the *ith* load step. Based on this response function, it is possible to determine the creep strain evolution process under any given load condition.

## 3. Results and discussion

### 3.1. Compressive fracture experimental results

According to the research process proposed above, the first step is to determine the compressive strength of the material. Table 1 shows the experimental results for this parameter, from which the value of the coefficient of variation is 5.2%. According to the experimental standard, the value of this parameter should be no more than 20% [24]. Thus, the experimental results can be used for further analysis.

**Table 1:** Results of the compressive fracture experiments.

Case number	Compressive load (kN)	Compressive strength (MPa)
1	56.1	89.76
2	59.3	94.8
3	52.6	84.18
4	60.6	96.97
5	54.4	87.1
6	62.8	100.43
7	61.4	98.2
8	59.1	94.56
9	60.8	97.28
10	59.5	95.21
11	58.6	93.8

**Fig 7:**
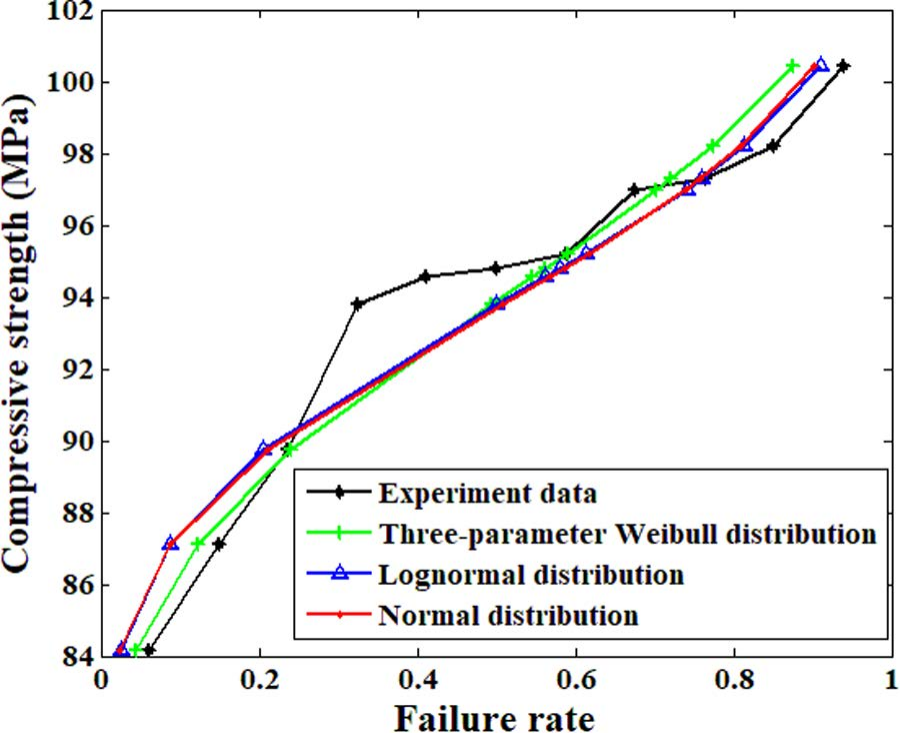
Statistical analysis results of the compressive strength.

The experimental results from different samples are quite different from each other; this makes the corresponding statistical analysis necessary. Fig 7 shows the analysis results based on the three selected functions. The fitting results based on these three functions are nearly the same (the relative difference at each failure rate is no more than 2%). However, the three-parameter Weibull function can determine a theoretical absolute safety point, which means that a stress level lower than this value will never result in fracture of the sample [25]. This advantage makes it more suitable for engineering design, especially in situations related to safety and reliability. Thus, the compressive strength of the material based on this function is 78.5 MPa.

### 3.2. Creep test results

**Fig 8:**
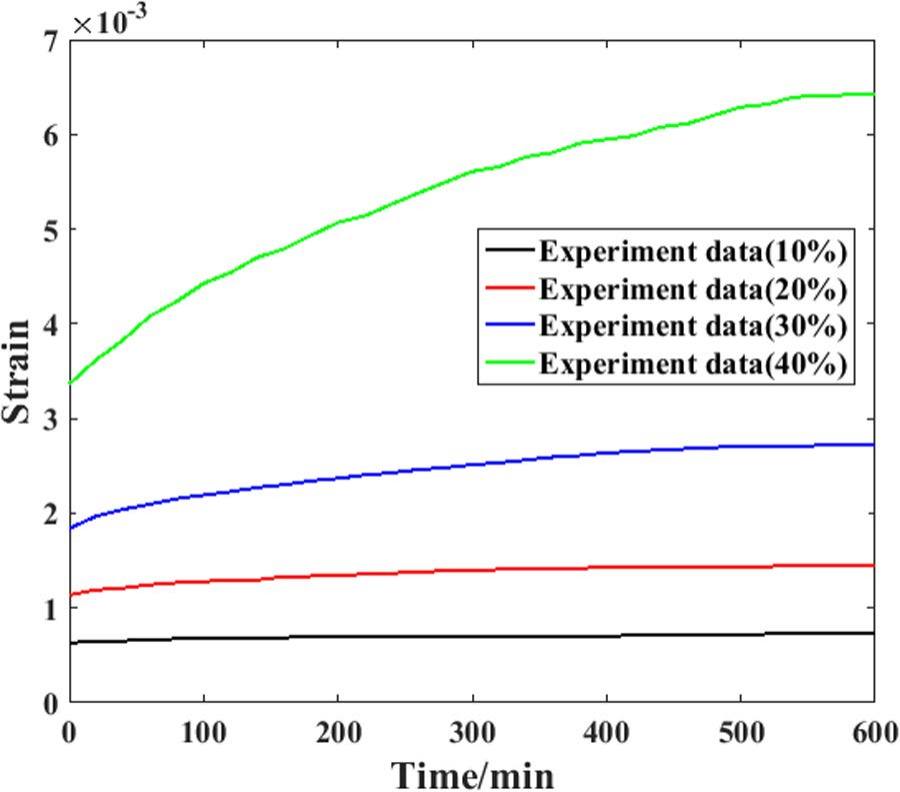
Creep evolution process of the first specimen.

**Fig 9:**
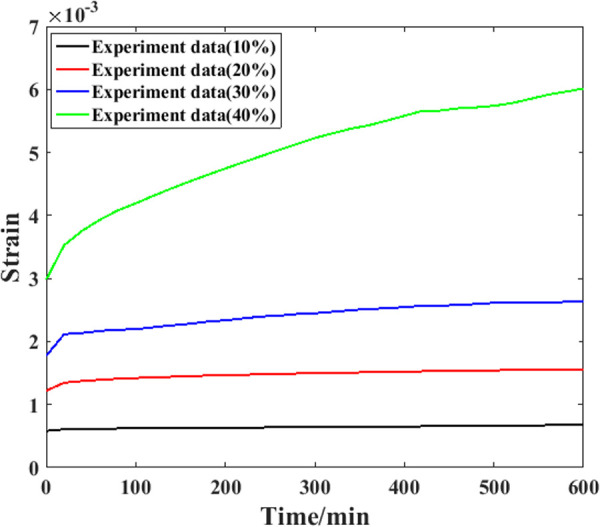
Creep evolution process of the second specimen.

**Fig 10:**
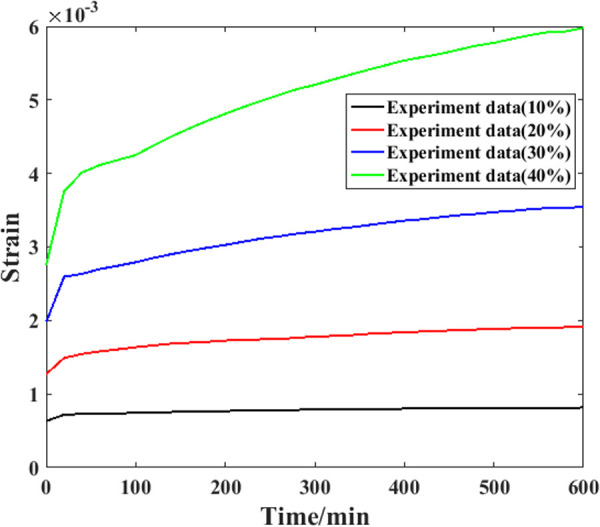
Creep evolution process of the third specimen.

Based on the compressive strength parameters determined above, it is possible to carry out corresponding creep experiments. However, to check the universality of the proposed model comprehensively, three samples were used for the tests in this study. Detailed information on the experimental results is shown in Figs 8–10.

As shown in Fig 8, for the first sample, when the stress level is relatively low (10% of the compressive strength), the compressive strain growth during the experimental process is not obvious, and the recorded curve of the strain is nearly parallel to the axial curve (the relative increment is 18%). Throughout the entire experimental process under this stress level, the growth speed of the creep strain is generally steady. When the stress level is greater (20% of the compressive strength), the slope of the creep strain curve is also obviously greater, which means that more compressive strain will be generated at this stress level. After approximately 100 min, the strain growth speed gradually decreases, and the curve slope is generally straight. For the creep strain recorded under the third stress level, the growth speed of the creep strain still increases with increasing stress level. After approximately 400 min, the growth speed gradually decreases, and the creep strain curve is generally straight. For the creep strain recorded under the fourth stress level, the growth speed is also the highest. In addition, the reduction point of the speed appears nearly at the end of the test. In general, when a higher stress level is applied to a sample, a faster increase in the creep strain will be generated, and the speed reduction point of the creep strain will appear later.

Fig 9 shows the experimental results recorded from the second sample. For this sample, the creep strain growth speed is also clearly enhanced by the stress level applied to it. In addition, for the creep strain recorded under the highest stress level, the growth speed becomes slower after approximately 410 minutes. After approximately 100 min, the growth speed increases again. This situation may result in the creep fracture of the sample. For the creep strain recorded from the third sample shown in Fig 10, similar results can be found. The main reason for this phenomenon can be attributed to the microstructure features of the sample [26]. In the manufacturing process of recombinant bamboo, the fiber of this material is bamboo fiber, and the matrix is phenolic resin mixed with the fiber. When an external compressive load is applied to a sample, both the fiber and the matrix can resist creep strain under relatively low stress levels. For the creep strain under higher stress levels, the difference between these two components results in greater shear stress at the interface between them. As a result, accumulated creep damage will result in the initiation of cracks within these areas, as well as stress concentration. These factors cause the sample to break.

### 3.3. Model analysis results and discussion

**Fig 11:**
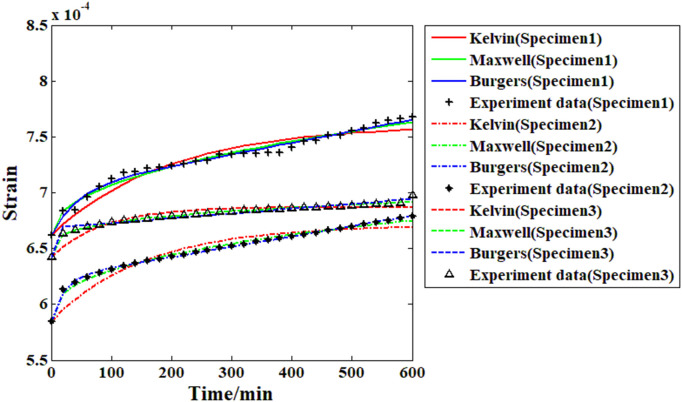
Fitting results of all the samples from the different models (under 10% stress level).

**Fig 12:**
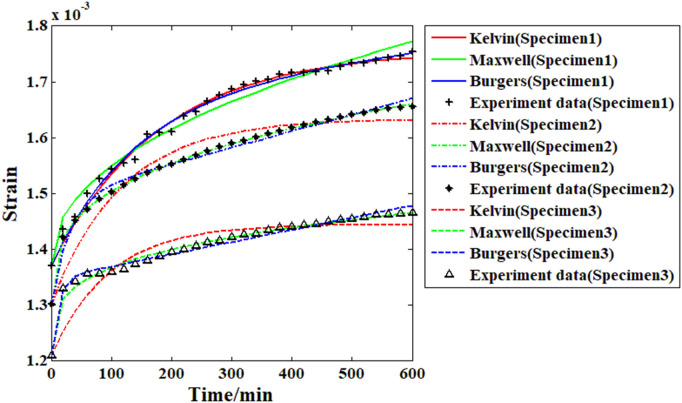
Fitting results of all the samples from the different models (under 20% stress level).

**Fig 13:**
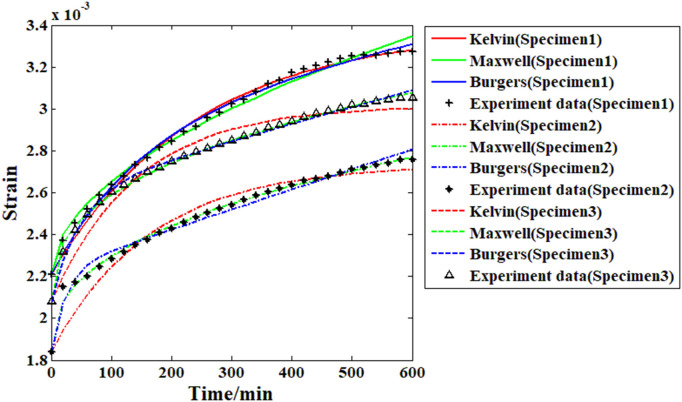
Fitting results of all the samples from the different models (under 30% stress level).

**Fig 14:**
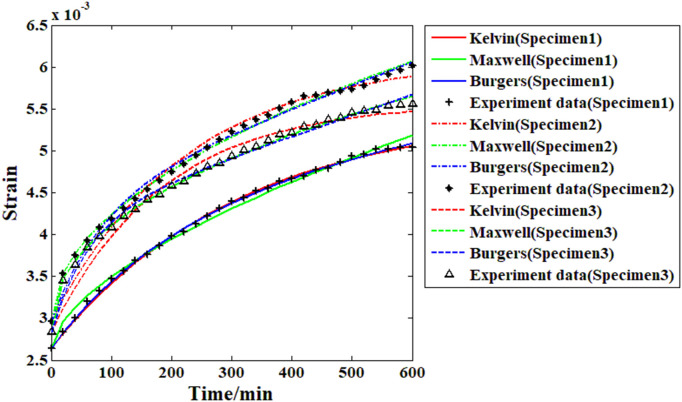
Fitting results of all the samples from the different models (under 40% stress level).

Based on the creep strain record results and the proposed Maxwell model, it is possible to analyze the viscoelastic behavior of the recombinant bamboo through the fitting approach. To analyze the viscoelastic properties of the material comprehensively, several other commonly used models (Burgers and Kelvin) are also adopted in the fitting approach for comparison. During the fitting process, the main parameters of the selected models are treated as independent variables, and the response of the creep strain is the corresponding dependent variable. The fitting goal is to minimize the relative difference between the actual experimental data and the response function as little as possible. The results are shown in Figs 11–14.

As shown in Fig 11, for the creep strain recorded under the first stress level (10% of the compressive strength), when the Kelvin model is applied in the fitting analysis, the results are quite different from the actual experimental data. For the Burgers model, the accuracy of the fitting results is obviously improved, especially for the first and second samples. However, for the third sample, the fitting result based on the Burgers model is still obviously different from the experimental data (the value of the correlation coefficient in this case is less than 0.96). For the proposed Maxwell model in this paper, the fitting results are quite close to the experimental data, which means that the accuracy of this model is much higher than those of the Kelvin and Burgers models. The analysis results in Fig 12 reveal that with increasing stress level, the accuracy of the fitting result based on the Kelvin model has improved to some degree but is still obviously far from the experimental data. For the Burgers model and the Maxwell model, the fitting results under this stress level are very close to the experimental data. For the analysis results under the 30% and 40% stress levels, the Burgers and proposed Maxwell models are still able to provide high enough accuracy. However, for the Kelvin model, the accuracy is still poor. In general, the Kelvin model is unable to provide satisfactory fitting results under these conditions. The Burgers model is quite useful for analyzing the compressive creep behavior of recombinant bamboo under relatively high stress levels. However, for the same behavior under a lower stress level, sometimes obvious errors may be found during the analysis, which restricts the corresponding application. For the proposed Maxwell model, which is defined based on the variable-order fractional derivative approach, the analysis results under all the stress levels are eligible for application. Thus, this model is more suitable for use in creep property research. The detailed information of the fitting results is shown in Table 2.

**Table 2:** Fitting results of the experimental data based on the variable-order fractional derivative-defined Maxwell model.

Specimen number	10% stress level	20%stress level	30% stress level	40% stress level
1	$\begin{array}{c} \eta_{0}=1582GPa\cdot \min^{-1}\\ \alpha =0.4272\\ R^{2}=0.9875\\ E_{0}=10.3GPa \end{array}$	$\begin{array}{c} \eta_{0}=1234GPa\cdot \min^{-1}\\ \alpha =0.4872\\ R^{2}=0.9879\\ E_{0}=10.2GPa \end{array}$	$\begin{array}{c} \eta_{0}=994GPa\cdot \min^{-1}\\ \alpha =0.5467\\ R^{2}=0.9951\\ E_{0}=9.92GPa \end{array}$	$\begin{array}{c} \eta_{0}=796GPa\cdot \min^{-1}\\ \alpha =0.6199\\ R^{2}=0.9962\\ E_{0}=9.8GPa \end{array}$
2	$\begin{array}{c} \eta_{0}=803GPa\cdot \min^{-1}\\ \alpha =0.297\\ R^{2}=0.9886\\ E_{0}=11.8GPa \end{array}$	$\begin{array}{c} \eta_{0}=555GPa\cdot \min^{-1}\\ \alpha =0.352\\ R^{2}=0.9963\\ E_{0}=10.7GPa \end{array}$	$\begin{array}{c} \eta_{0}=468GPa\cdot \min^{-1}\\ \alpha =0.4167\\ R^{2}=0.9924\\ E_{0}=10.4GPa \end{array}$	$\begin{array}{c} \eta_{0}=356GPa\cdot \min^{-1}\\ \alpha =0.499\\ R^{2}=0.9981\\ E_{0}=11.2GPa \end{array}$
3	$\begin{array}{c} \eta_{0}=320GPa\cdot \min^{-1}\\ \alpha =0.281\\ R^{2}=0.9966\\ E_{0}=10.6GPa \end{array}$	$\begin{array}{c} \eta_{0}=241GPa\cdot \min^{-1}\\ \alpha =0.325\\ R^{2}=0.9972\\ E_{0}=10.7GPa \end{array}$	$\begin{array}{c} \eta_{0}=196GPa\cdot \min^{-1}\\ \alpha =0.37\\ R^{2}=0.9966\\ E_{0}=11GPa \end{array}$	$\begin{array}{c} \eta_{0}=182GPa\cdot \min^{-1}\\ \alpha =0.404\\ R^{2}=0.9977\\ E_{0}=10.9GPa \end{array}$

As shown in Table 2, the fitting results based on the proposed Maxwell model are quite similar to the exact experimental data (the R^2^ values in all the cases are greater than 0.98). In addition, for every sample, the value of the order increases with increasing stress level. For the coefficient of viscosity of the dashpot body, the situation is completely the opposite. According to the definition of the Maxwell model and the theory of the fractional derivative, with increasing stress level, the material shows more rheological properties; this is in accordance with the creep strain growth speed. According to the definition of the Burgers model, there are four parameters. For the proposed Maxwell model in this paper, only three parameters are adopted in building the model. This feature can be treated as another advantage in the popularization and application of the model.

### 3.4. Model parameter analysis results

**Fig 15:**
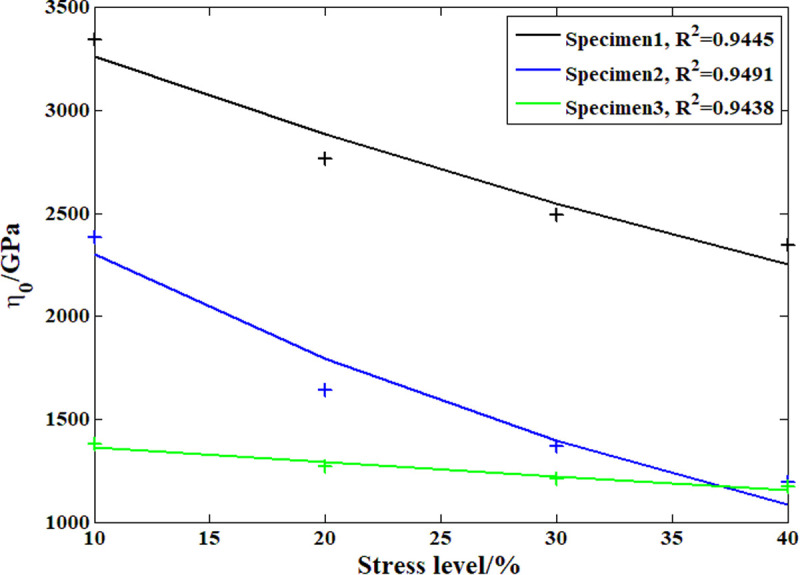
Analysis results of the order based on the power function.

**Fig 16:**
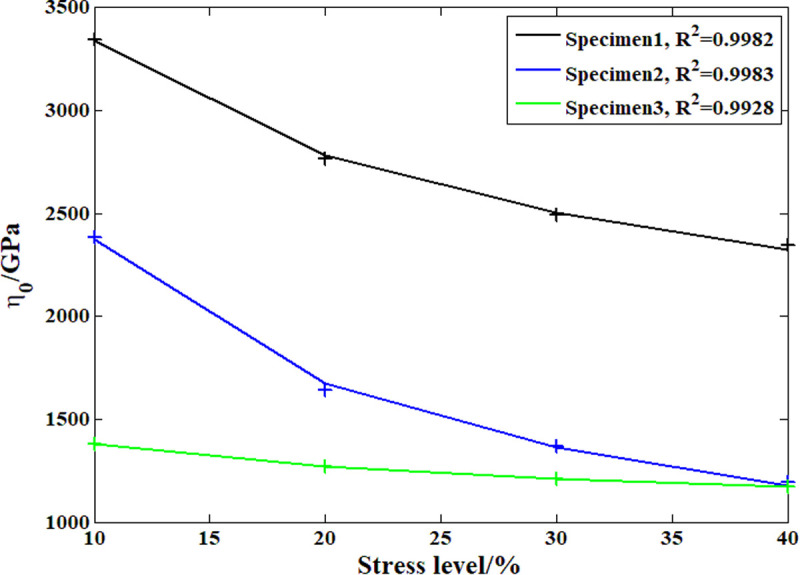
Analysis results of the order based on the exponential function.

**Fig 17:**
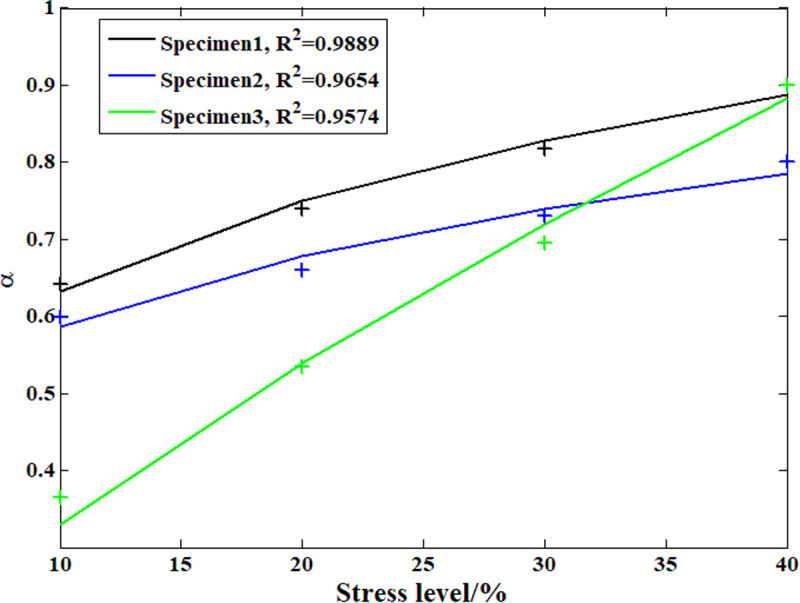
Analysis results of the viscosity coefficient based on the power function.

**Fig 18:**
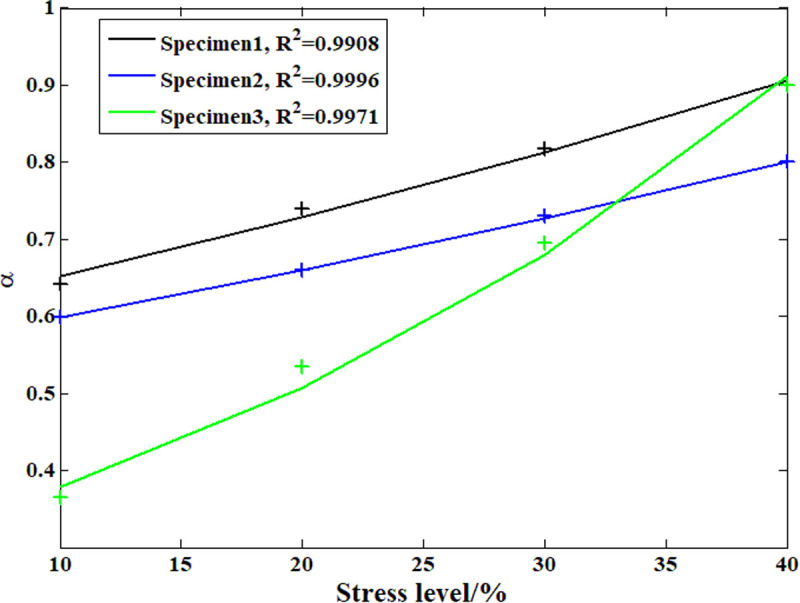
Analysis results of the viscosity coefficient based on the exponential function.

According to the analysis above, during the compressive creep process of the recombinant bamboo, the stress level clearly affects the creep behavior. In previous studies, the elastic and plastic properties of recombinant bamboo under different stress levels were studied in detail with corresponding empirical models. However, studies on the effect of stress level on the viscoelastic properties of recombinant bamboo have rarely been reported. On the other hand, according to the definition of the Maxwell model, the viscoelastic property is provided by the dashpot body. Figs 15–18 show the analysis results of the stress level effect on the main parameters of the dashpot body defined by the variable-order fractional derivative approach. Clearly, for the order of the model, the exponential function can express the relationship between the stress level and this parameter more accurately than the power function, which is suitable for application under these conditions. For the coefficient of viscosity, the opposite is the case. Based on these relationships, it is possible to predict the creep behavior of the same sample under any other stress level.

**Fig 19:**
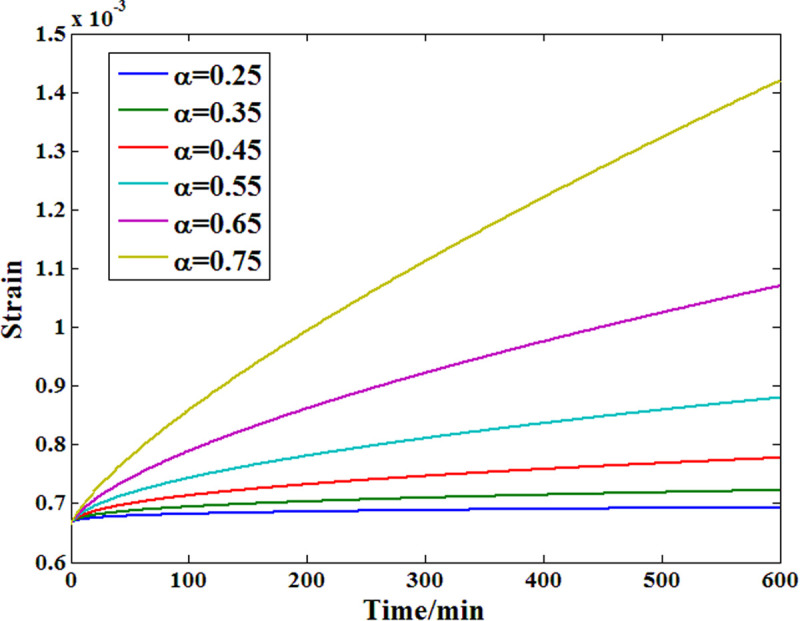
Parameter sensitivity analysis results of the order.

**Fig 20:**
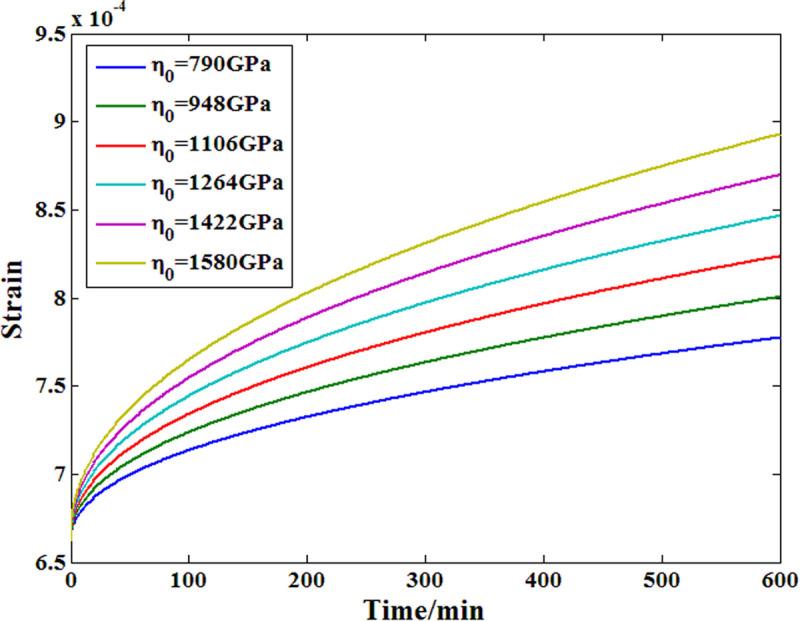
Parameter sensitivity analysis results of the viscosity coefficient.

To further analyze the feasibility of the proposed Maxwell model in researching the viscoelastic behavior of a material, a corresponding model parameter sensitivity analysis was also conducted. Figs 19 and 20 show the results of the two main parameters, among which the value of the order has a positive effect on the creep strain growth rate, as does the viscosity coefficient. In addition, compared with the viscosity coefficient of the dashpot body, the order affects the strain growth speed more obviously within the definition range.

## 4. Conclusions and further work plan

Fiber-reinforced composite materials, such as recombinant bamboo, usually exhibit obvious creep properties during their working process. In this work, a detailed study was conducted on the compressive creep performance of this material. A time-varying compressive load is applied to different samples to record the creep strain evolution process. In addition, different kinds of mechanical constitutive models were selected to analyze the viscoelastic properties of the material. The main conclusions drawn from this study are as follows:

1) When an external compressive load is applied to the recombinant bamboo, the creep growth is obviously influenced by the stress level of the load condition. When a higher stress level is applied, a faster creep strain growth speed will be generated, and a later speed drop point will appear, which can be explained by the microstructure features of the material.
2) The proposed Maxwell model can accurately simulate the compressive creep strain growth property of all the samples under all the stress levels, which makes it superior to the other two selected models in researching this problem. In addition, the stress level effect on the main model parameters can be expressed by the selected functions, which makes the prediction of the creep strain evolution under other stress levels possible.
3) The main advantage of the proposed model is that the numbers of the model parameters is relatively less, which makes it easier for the popularization. In addition, the accuracy provided by this model is also the highest. These advantages makes this model valuable for the application.

In this paper, the research object is the compressive creep performance of the recombinant bamboo. For other kinds of similar composite materials such as the laminated wood, the feasibility of the proposed model is unclear. In addition, the time period of the creep experiment process carried out in this paper is relatively short. Thus whether the model is suitable for researching the long term creep performance of the material is also unclear. On the other hand, the creep process of the viscoelastic material is usually associated with the accumulation of the deformation and damage. Thus more work should be conducted on the damage mechanism of the material and the relationship between it with the macroscopical mechanical property.

## Supporting information

S1 File Strain. (XLSX)
